# NOD1 Activation Induces Cardiac Dysfunction and Modulates Cardiac Fibrosis and Cardiomyocyte Apoptosis

**DOI:** 10.1371/journal.pone.0045260

**Published:** 2012-09-18

**Authors:** María Fernández-Velasco, Patricia Prieto, Verónica Terrón, Gemma Benito, Juana M. Flores, Carmen Delgado, Carlos Zaragoza, Begoña Lavin, Mónica Gómez-Parrizas, Eduardo López-Collazo, Paloma Martín-Sanz, Lisardo Boscá

**Affiliations:** 1 TumorImmunology Lab, Instituto de Investigación Hospital La Paz, Madrid, Spain; 2 Instituto de Investigaciones Biomédicas Alberto Sols (Centro Mixto Consejo Superior de Investigaciones Científicas-UAM), Madrid, Spain; 3 Departamento de Medicina y Cirugía Animal, Facultad de Veterinaria, Universidad Complutense de Madrid, Madrid, Spain; 4 Centro de Investigaciones Biológicas, Consejo Superior de Investigaciones Científicas. Madrid, Spain; 5 Departamento de Farmacología, Facultad de Medicina, Universidad Complutense de Madrid, Madrid, Spain; 6 Centro Nacional de Investigaciones Cardiovasculares, Madrid, Spain; 7 Centro de Investigación Biomédica en Red de Enfermedades Hepáticas y Digestivas, Barcelona, Spain; University of Western Ontario, Canada

## Abstract

The innate immune system is responsible for the initial response of an organism to potentially harmful stressors, pathogens or tissue injury, and accordingly plays an essential role in the pathogenesis of many inflammatory processes, including some cardiovascular diseases. Toll like receptors (TLR) and nucleotide-binding oligomerization domain-like receptors (NLRs) are pattern recognition receptors that play an important role in the induction of innate immune and inflammatory responses. There is a line of evidence supporting that activation of TLRs contributes to the development and progression of cardiovascular diseases but less is known regarding the role of NLRs. Here we demonstrate the presence of the NLR member NOD1 (nucleotide-binding oligomerization domain containing 1) in the murine heart. Activation of NOD1 with the specific agonist C12-iEDAP, but not with the inactive analogue iE-Lys, induces a time- and dose-dependent cardiac dysfunction that occurs concomitantly with cardiac fibrosis and apoptosis. The administration of iEDAP promotes the activation of the NF-κB and TGF-β pathways and induces apoptosis in whole hearts. At the cellular level, both native cardiomyocytes and cardiac fibroblasts expressed NOD1. The NLR activation in cardiomyocytes was associated with NF-κB activation and induction of apoptosis. NOD1 stimulation in fibroblasts was linked to NF-κB activation and to increased expression of pro-fibrotic mediators. The down-regulation of NOD1 by specific siRNAs blunted the effect of iEDAP on the pro-fibrotic TGF-β pathway and cell apoptosis. In conclusion, our report uncovers a new pro-inflammatory target that is expressed in the heart, NOD1. The specific activation of this NLR induces cardiac dysfunction and modulates cardiac fibrosis and cardiomyocyte apoptosis, pathological processes involved in several cardiac diseases such as heart failure.

## Introduction

The innate immune system is responsible for the initial response of an organism to potentially harmful stressors, e.g. pathogens or tissue injury, and accordingly plays an essential role in the pathogenesis of many inflammatory diseases [1]. Recent studies have established a relationship between the innate immune response and some cardiovascular diseases [2]. Indeed, enhanced TLR signaling [3] has been detected in various cardiac pathologies including coronary heart disease, myocardial infarction and diabetes [4], [5], [6]. To this regard, it has been suggested that TLR-induced inflammation contributes to the development of acute coronary syndromes in patients with coronary artery disease [7]. Moreover, increased TLR4 expression has been detected in the hearts of patients with advanced heart failure [8], [9].

**Figure 1:**
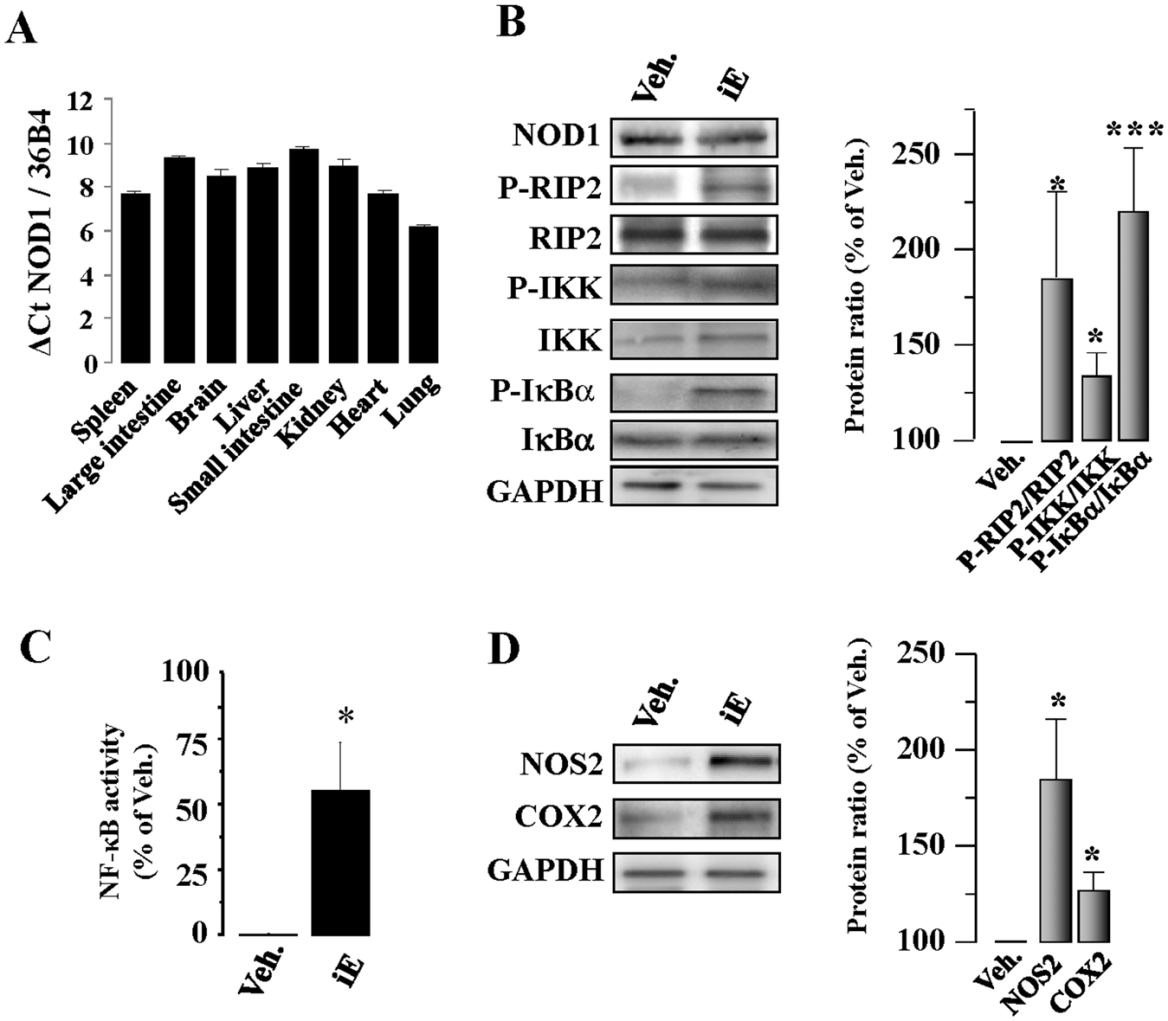
Murine cardiac tissue expresses NOD1. Specific stimulation of NOD1 induces NF-κB pathway activation. (A) Histograms show NOD1 mRNA levels in different mouse tissues. (B) NOD1 protein levels in mouse hearts were analyzed by western-blot. Animals received i.p. 150 µg of iEDAP (iE)/day, a selective agonist of NOD1, or vehicle (Veh.). After 2 weeks of treatment an up-regulation of P-RIP2/RIP2, P-IKK/IKK, P-IκBα/IκBα protein ratio (B), higher p65 binding to κB motifs determined by ELISA (C), and NOS2 and COX2 (D) protein levels were observed in iE treated hearts. NOS2 and COX2 protein values were normalized with GAPDH. Representative blots are shown in the left panels and right panels illustrate the histograms representing the mean (band ratio)±SEM values *vs.* Veh. (100%); n = 4–6 animals. *p<0.05, ***p<0.001 *vs.* vehicle.

Besides TLR, the innate response includes a family of cytoplasmic receptors that recognize components of microorganisms and abnormal/damaged host cells: the nucleotide-binding oligomerization domain-like receptors (NLRs) [10]. NLRs activation promotes a cascade of molecular signaling events that ultimately lead to inflammation and cell death [11]. In humans, the NLR family is composed of 23 members that share a central NACHT domain and a carboxy-terminal leucine-rich repeat region [12]. NOD1 (nucleotide-binding oligomerization domain containing 1) is a NLR member that contains an amino terminal caspase recruitment domain (CARD) required for triggering nuclear factor-κB (NF-κB) signaling. Activation of NOD1 involves the formation of a multiprotein signaling complex that includes RICK (RIP2) and initiates a variety of cellular responses including NF-κB activation, cytokine production and apoptosis induction [13], [14], [15], [16]. NOD1 is widely expressed in many cell types including epithelial and dendritic cells [17], [18]. To date, no data have been published concerning the presence of NOD1 in the heart, and the possible effects of its activation in cardiac cells. In the present study, we identify the presence of this NLR in mouse hearts. The specific stimulation of NOD1 with C12-iEDAP (iE) induces cardiac dysfunction that occurs concomitantly with cardiac fibrosis and apoptosis; however, iE-Lys, a chemically related molecule to iE but lacking NOD1 activation capacity, failed to exert cardiac dysfunction, pro-fibrotic or apoptotic effects. Administration of a NOD1 agonist (iE) induces the activation of NF-κB, TGF-β and apoptotic signaling pathways in whole hearts. At the cellular level, both cardiomyocytes and cardiac fibroblasts expressed NOD1. NLR activation in cardiomyocytes was associated with NF-κB pathway activation and with induction of apoptosis, whereas in fibroblasts was linked to NF-κB pathway activation and to increased expression of pro-fibrotic mediators. The down-regulation of NOD1 by specific siRNAs blunted the effect on the pro-fibrotic TGF-β pathway and cell apoptosis induced after selective NOD1 activation. Collectively, our results indicate that NOD1 stimulation induces cardiac dysfunction and modulates cardiac fibrosis and cardiomyocyte apoptosis, pathological processes involved in several diseases such as heart failure.

**Figure 2:**
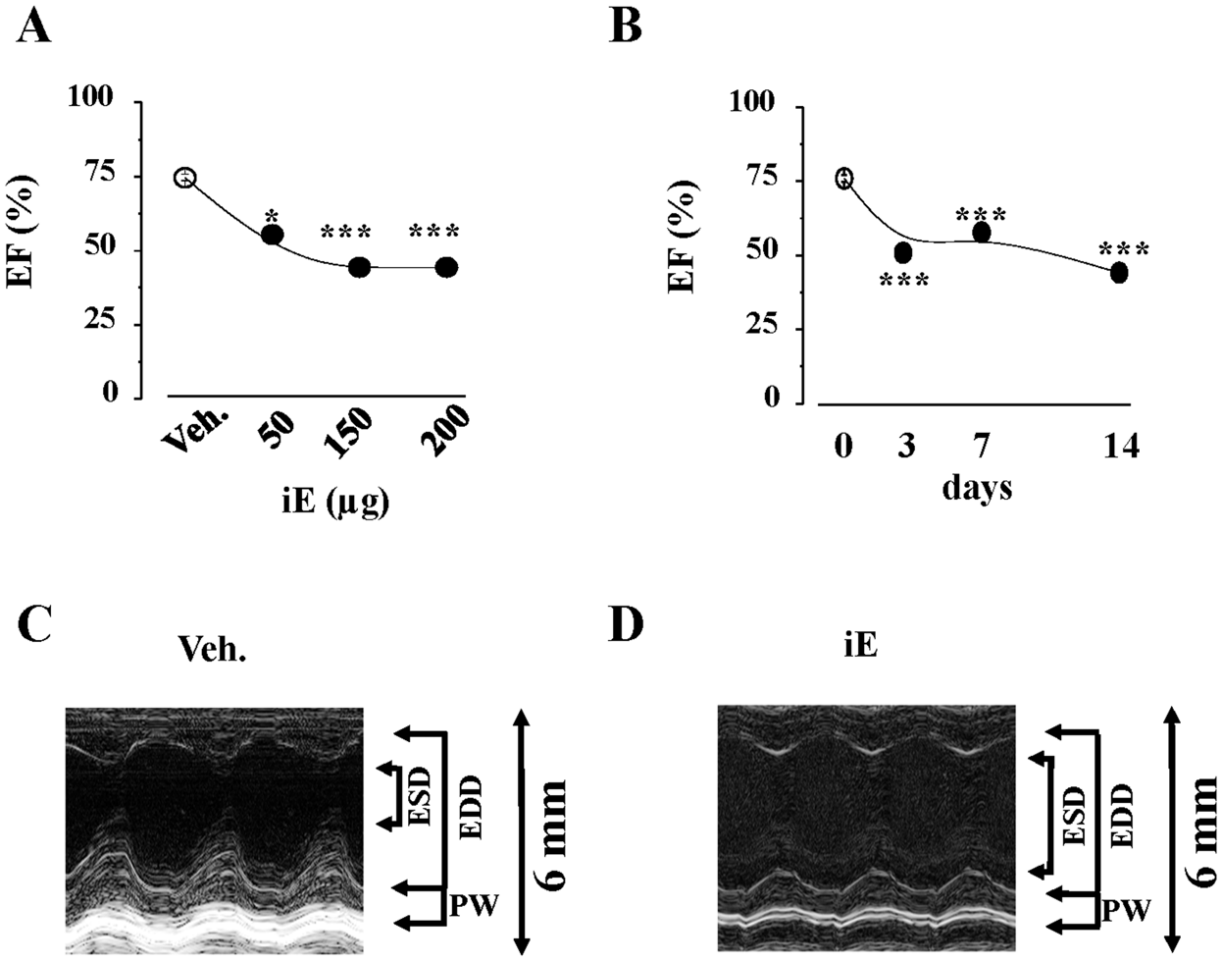
Specific activation of NOD1 promotes cardiac dysfunction. (A) Ejection fraction obtained in mice treated for 2 weeks (daily) with vehicle or the indicated concentrations of iE. (B) Ejection fraction obtained in mice treated during 3, 7 or 14 days with vehicle or 150 µg of iE. (C,D) Representative echocardiographic images (M mode) of mouse hearts treated for two weeks with vehicle (C) or 150 µg of iE (D). End Systolic Diameter (ESD), End Diastolic Diameter (EDD), and left ventricle Posterior Walls (PW) are indicated. *p<0.05, ***p<0.001 *vs.* vehicle.

## Materials and Methods

### Chemicals

To selective stimulate NOD1 the agonist C12-iE-DAP (iE, InvivoGen) was used [19], [20]. Animals received i.p. 150 µg of iE, or vehicle (Veh.) per day for a total of 2 weeks [21], [22]. iE-Lys was used as negative control of C12-iE-DAP (InvivoGen). Staurosporine was from Calbiochem (San Jose, CA).

**Table 1:** Cardiac parameters collected after M-mode ultrasound evaluation of mice.

Parameter	Treatment				
	Veh.	iE 50	iE 150	iE 200	iE-Lys
**BW (g)**	28.9±0.7	28.9±0.8	27.7±0.3	28.5±0.6	26.5±0.8
**HW (mg)**	222.6±10.9	226.7±28.4	223.8±16.6	233.5±20.5	197.0±26.0
**HR (bpm)**	442.8±8.7	460.8±1.3	437.8±16.8	409.6±4.7*	437.2±4.5
**EF (%)**	74.6±1.1	55.4±1.7*	44.4±0.4***	44.4±0.9***	69.2±9.6
**FS (%)**	38.0±1.5	28.3±0.9***	22.1±0.9***	25.5±2.7***	39.1±7.6
**LVESD** **(mm)**	2.2±0.1	2.3±0.2	2.8±0.1	2.8±0.3	2.3±0.5
**LVEDD** **(mm)**	3.8±0.1	3.8±0.1	3.8±0.1	3.9±0.3	3.8±0.4
**SV (mL)**	16.4±0.9	18.4±1.5	22.8±1.9***	28.4±2.4***	20.1±10.6
**DV (mL)**	60.3±4.2	63.6±2.1	60.9±2.9	64.8±12.7	62.3±15.3
**N**	6	4	4	6	3

Data are means ± SE. HW, heart weight, BW, body weight, HR heart rate, EF, left-ventricle ejection fraction, FS, fractional shortening, LVESD, left-ventricle end-systolic diameter; LVEDD, left-ventricle end-diastolic diameter; SV, systolic volume; DV, diastolic volume;

** *p*<0.01,

*** *p*<0.001 Vehicle (Veh.) *vs.* iE (50, 150 or 200 µg iEDAP treated mice for two weeks).

### Animal Care and Cell Isolation

The study was conducted following the guidelines of the Spanish Animal Care and Use Committee, according to the guidelines for ethical care of experimental animals of the European Union (2010/63/EU). This study conforms to the Guide for the Care and Use of Laboratory Animals published by the U.S.National Institute of Health (NIH Publication No. 85–23, revised 1996). All procedures were approved by the Bioethical Committee of the Consejo Superior de Investigaciones Científicas and Autonomous University of Madrid. Animals were injected with buprenorfin (0.01 mg/kg, i.p.) and 30 min later, mice were anesthetized with sodium pentobarbital (100 mg/kg, i.p.) and heparin. Hearts from adult mice were rapidly removed and ventricular cardiomyocytes were isolated from C57BL/6J mice using a standard enzymatic digestion as previously described [23]. Fibroblasts resent in the adult murine hearts were isolated from the supernatant obtained during the cardiomyocyte isolation. Fibroblasts were collected by centrifugation (300 ***g***, 10 min) and seeded in Dulbecco’s modified Eagle’s medium (DMEM, Gibco) containing 100 U/ml penicillin, 100 mg/ml streptomycin, 15% heat-inactivated FBS and 50 ng/ml of insulin/transferrin/selenium (ITS, Sigma). Isolated fibroblasts expressed markers of fibroblastic lineage, such as vimentin and DDR2 [24], [25] and showed negligible labeling with actin.

**Figure 3:**
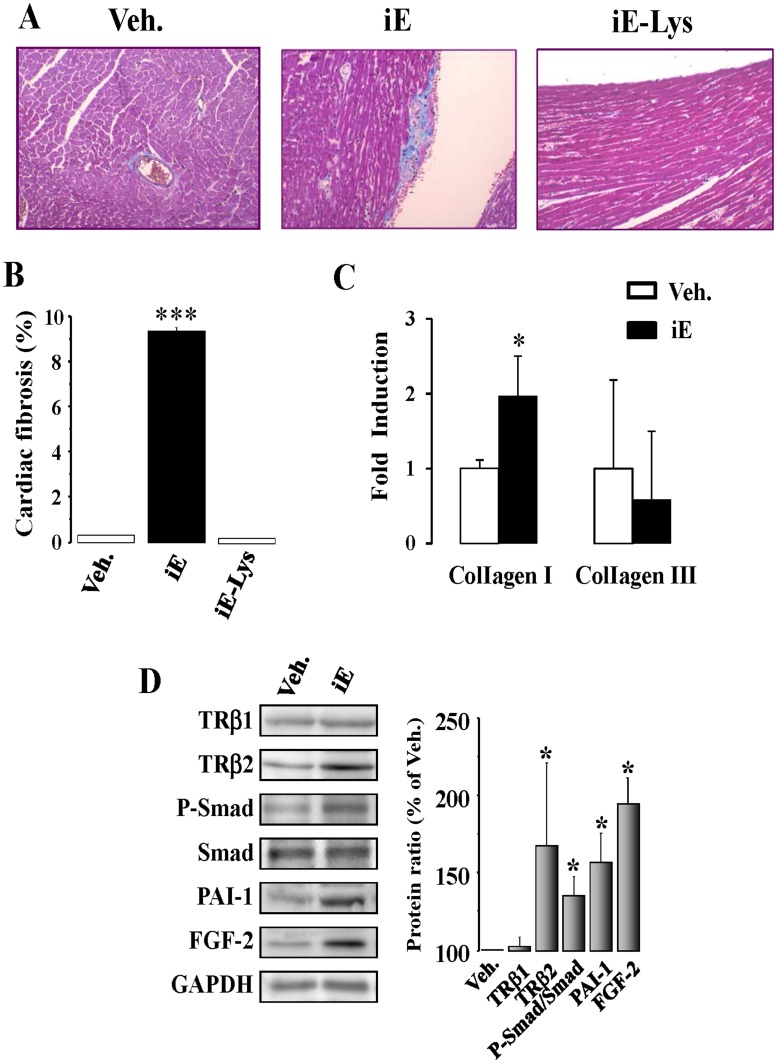
NOD1 activation increases cardiac fibrosis and TGF-β pathway in hearts isolated from iE treated mice. (A) Histological analysis of the hearts from mice treated with vehicle (Veh.), with 150 µg of iEDAP (iE) or with 150 µg of iE-Lys (NOD1 inactive analogue of iEDAP). Representative whole-heart cross-sections are shown (magnification x20). Myocardial fibrosis was evaluated after staining with Masson’s trichrome. (B) Histograms show the quantitative analysis of subendocardial fibrosis is shown as collagen area *vs.* total tissue area (100%, mean ± SEM; n = 4 animals per condition). (C) Histograms show type I and III collagen mRNA levels in cardiac tissue from iE and Veh. treated mice. (D) TGF-β signaling was activated in hearts from iE-treated mice. *Left panel*, representative blots of TRβ1, TRβ2, P-Smad, Smad, PAI-1 and FGF-2 of vehicle and iE treated mouse hearts (150 µg for 2 weeks). *Right panel,* shows the corresponding histograms representing the mean values *vs.* vehicle (100%, n = 4–6 animals). GAPDH was used to normalize all the target protein levels. *p<0.05, ***p<0.001 *vs.* vehicle.

**Figure 4:**
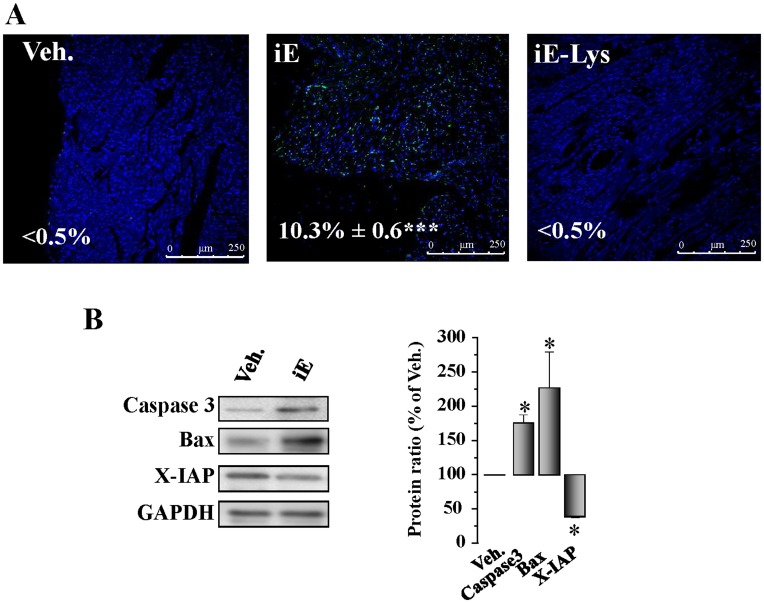
Selective stimulation of NOD1 induces cardiac apoptosis. Animals received i.p. 150 µg/day of iEDAP (iE), 150 µg/day of iE-Lys or vehicle for 2 weeks. (A) TUNEL staining of cells undergoing apoptosis in cardiac tissue sections from vehicle, iE or iE-Lys-treated mice. Light transmission of the preparations indicated that apoptotic cells were predominantly cardiomyocytes. Representative images of TUNEL positive (green) and DAPI (blue) staining (magnification x 40). The percentages of positive TUNEL cells are indicated in the images. (B) Caspase 3, Bax and X-IAP levels determined by Western blot from hearts of vehicle and iE mice. *Left panel* shows the histograms representing the mean±SEM *vs.* vehicle (100%). *p<0.05, ***p<0.001 *vs.* vehicle; n = 4–6 animals.

### M-mode Echocardiography

Mouse hearts were visualized by echocardiography over time, using a high-frequency micro-ultrasound system (Vevo 770; VisualSonics, Toronto, Canada). During all experiments, mice were anesthetized with 1.5% isoflurane gas, which resulted in a heart rate of *ca.* 300 beats/min. Anesthetized animals were placed on the Integrated Rail System and Mouse Handling Table (provided by the manufacturer), which allows simultaneous acquisition of temperature data (37°C, by using the integrated heating pad). The chest of the mouse was carefully shaved, and warm ultrasound transmission gel was applied to ensure optimal image quality. Parasternal short-axis-view images of the heart were recorded using a 30-MHz RMV scan head in a B-mode to allow M-mode recordings by positioning the cursor in the parasternal short-axis view perpendicular to the interventricular septum and posterior wall of the left ventricle. From these recordings, the following parameters were determined using the on-site software cardiac package (VisualSonics): heart rate, left-ventricle ejection fraction, fractional shortening, left-ventricle end-systolic diameter, left-ventricle end-diastolic diameter, systolic volume and diastolic volume.

### RNA Isolation and RT-PCR Analysis

A total of 1 µg total RNA, extracted with TRIzol reagent (Invitrogen) according to the manufacturer’s instructions, was reverse transcribed using Transcriptor First Strand cDNA Synthesis Kit for RT-PCR following the indications of the manufacturer (Roche). Real-time PCR was conducted on a MyiQ Real-Time PCR System (Bio-Rad) using Taqman Gene Expression Assay (Applied Biosystem). PCR thermocycling parameters were 50°C for 2 min, 40 cycles of 95°C for 15 s, and 60°C for 1 min. NOD1 and the housekeeping 36B4 expression were analyzed in parallel in all samples. For type I and type III collagen, ANP and β-MHC amplification reaction using Quanti SYBR Green RT protocol (Roche) was performed. Melting curve data were collected to check the RT-PCR specificity. Each cDNA was amplified in triplicate and the corresponding sample without reverse transcriptase (no-RT sample) was included as negative control. The expression of the chosen genes was normalized to that of 36B4. The replicates were then averaged, and fold induction was determined in a ΔΔCt-based fold-change calculations.

Primers were used for type I collagen (forward primer 5′-AAT GGC ACG GCT GTG TGC GA-3′, reverse primer 5′- AGC ACT CGC CCT CCC GTC TT-3′), type III collagen (forward primer 5′-CTG TAA CAT GGA AAC TGG GGA AA-3′, (reverse primer 5′-CCA TAG CTG AAC TGA AAA CCA CC-3′), ANP (forward primer 5′-ATT GAC AGG ATT GGA GCC CAG AGT-3′, reverse primer 5′-TGA CAC ACC ACA AGG GCT TAG GAT-3′), β-MHC (forward primer 5′-TCT ACA GGC CTG GGC TTA CCT CTC T-3′, (reverse primer 5′-ACT TCC GCA GGA AGG GGG CT-3′), and 36B4 was used as a house-keeping gene to normalize relative mRNA target levels (forward primer 5′-AGA TGC AGC AGA TCC GCA T-3′), (reverse primer 5′-GTT CTT GCC CAT CAG CAC C-3′).

**Figure 5:**
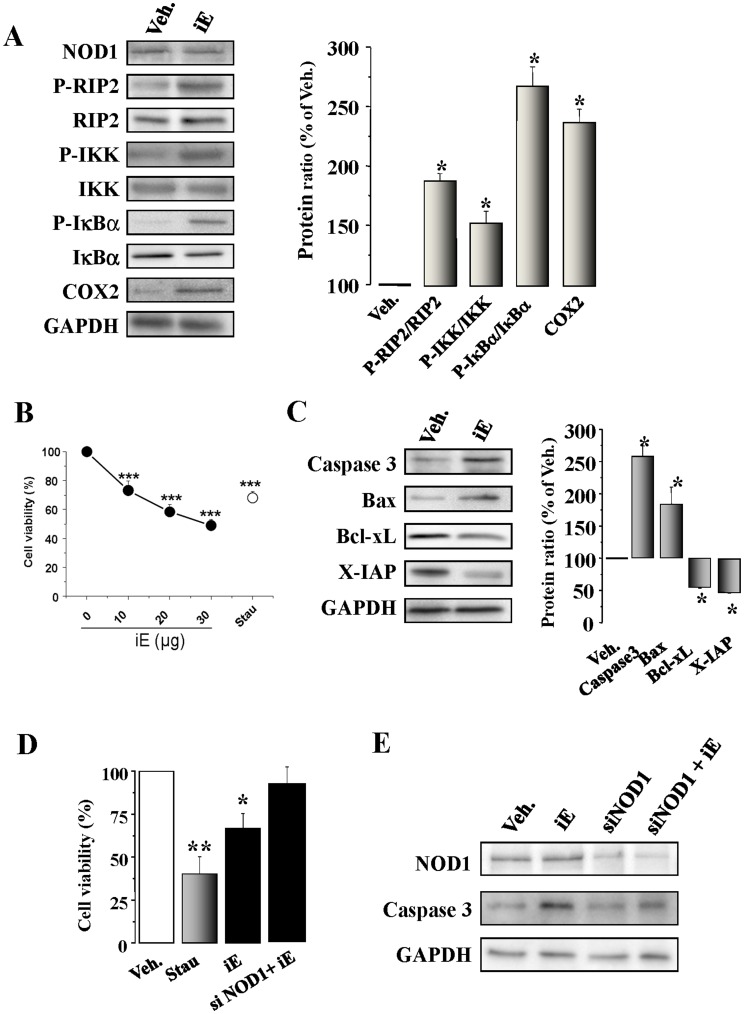
The NOD1 agonist iE induces NF-κB activation and apoptosis in cardiomyocytes and H9c2 cells. Effect of NOD1 ablation with siRNAs. (A) Native murine cardiomyocytes were incubated for 15 to 60 min, or 48 h with 20 µg/ml of iE. iE treatment induces an up-regulation of P-RIP2/RIP2, P-IKK/IKK, P-IκBα/IκBα (15–60 min) and COX2 (48 h) protein levels. (B) iE induces a decrease in cardiomyocyte viability in a dose-response form. Cardiomyocytes incubated for 2 h with 10–30 µg/ml of iE; staurosporine (Stau, 100 ng/ml) were used as a positive control to induce apoptosis. (C). iE treatment induces an up-regulation of caspase 3 and Bax protein levels and down-regulates the BcL-xL and X-IAP protein levels. (D) iE or Stau administration for 24 h in H9c2 promoted a decrease in viability as determined by MTT activity. NOD1 suppression by siRNA prevented the effect of iE on cell viability. (E) Representative blot of NOD1, caspase 3 and GAPDH obtained in vehicle and iE treated cells, NOD1 siRNAs (siNOD1) and NOD1 siRNA+iE in H9c2 cells. All targets protein levels were normalized by GAPDH. Data are expressed in histograms representing the mean±SEM *vs*. vehicle (100%); n = 3–5 samples per condition.*p<0.05, **p<0.01 and ***p<0.001 *vs.* vehicle.

### Preparation of Total Protein Cell Extracts

Tissues/cells were homogenized using a hand-held blender in a lysis buffer (in mM: 50 Tris, 320 sucrose and 1 DTT, pH 7.0, plus a complete protease and phosphatase inhibitor solutions, Sigma). The homogenate was spun at 13000 ***g*** for 10 min at 4°C and supernatants were frozen and stored at −80°C for Western blot analysis. Protein concentrations were determined by the Bradford assay (Bio-Rad).

### Preparation of Nuclear and Cytosolic Protein Extracts

Cardiac tissue was homogenized by 10 s sonication at 4°C in homogenization cytosolic buffer (in mM: 10 HEPES; pH 8.0, 10 KCl, 1 EDTA, 1 EGTA and 0.5% NP-40). Tissue pieces were vortexed and centrifuged at 12000 ***g*** for 30 min. The supernatant was the cytosolic fraction. The pellet was resuspended in 50 µl of ice-cold nuclear buffer (in mM: 20 HEPES; pH 8.0, 0.4 NaCl, 1 EDTA, 1 EGTA and 20% glycerol) and vortexed at 4°C for 30 min. After centrifugation (12000 ***g*** at 4°C for 20 min), the supernatant (nuclear fraction) was collected. All buffers contained a protease and phosphatase inhibitor cocktail (Sigma).

### Western Blot Analysis

Equal amounts of protein (20–80 µg) were loaded onto a 10–12% SDS-PAGE gel. Proteins were size fractionated, transferred to a Hybond-P membrane (GE Healthcare) and, after blocking with 5% nonfat dry milk, incubated with the corresponding antibodies. The blots were developed by the ECL protocol (GE Healthcare) and different exposure times were performed for each blot with a charge-coupled device camera in a luminescent image analyzer (Molecular Imager, Bio-Rad) to ensure the linearity of the band intensities. Values of densitometry were determined using Quantity One software (Bio-Rad). Antibodies for NOD1, P-RIP2, RIP2, P-IKK, IKK, P-IκBα, IκBα, NOS2, COX2, TRβ1, TRβ2, P-Smad (Smad 2/3), Smad, PAI-1, Bcl-xL, Bax, Bad, X-IAP and FGF-2 were from Santa Cruz Biotech or Cell Signaling Technology.

**Figure 6:**
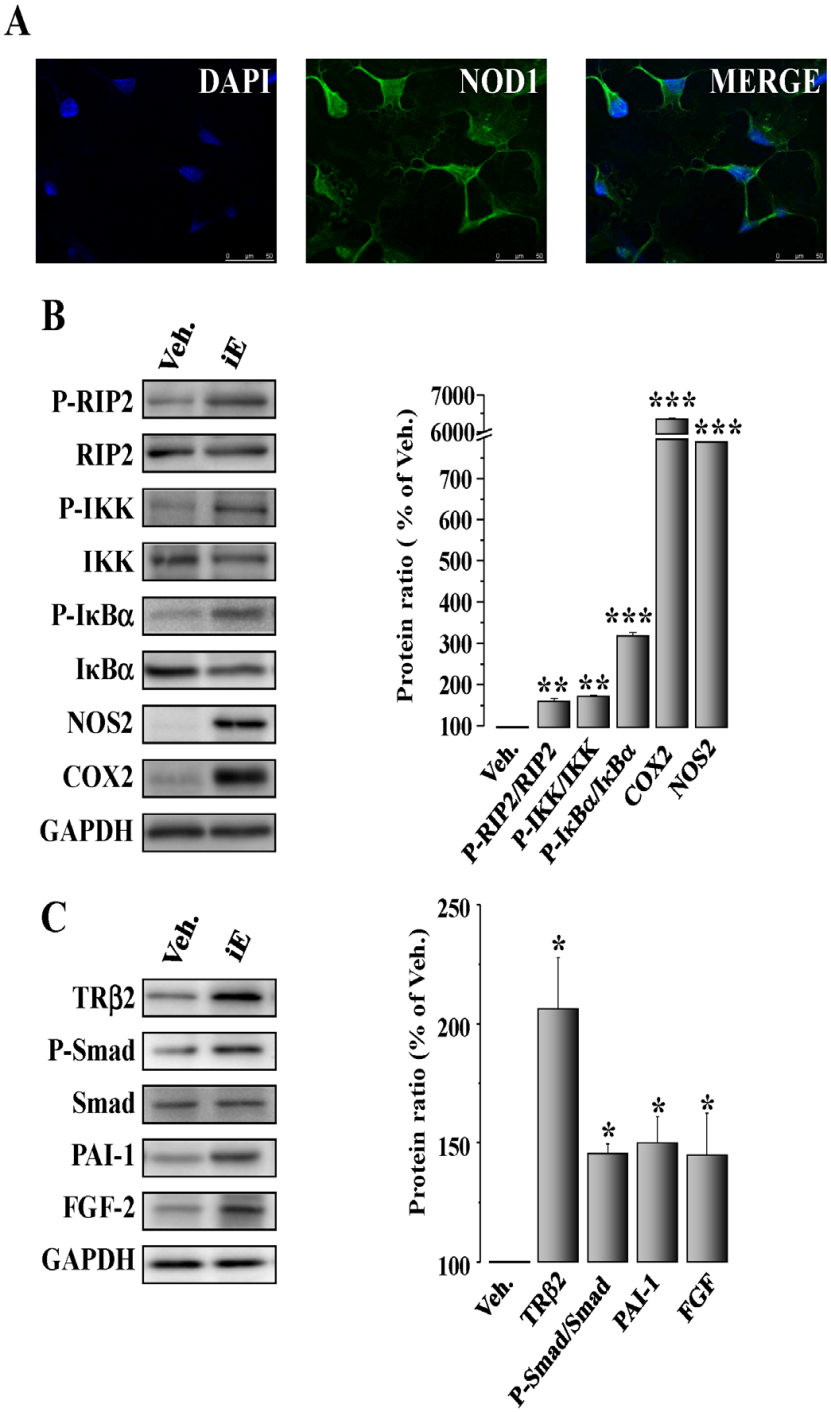
Selective activation of NOD1 stimulates NF-κB and TGF-β pathways in cardiac fibroblasts. (A) Confocal microscopy images of cardiac fibroblasts isolated from mouse hearts stained for NOD1. (B) Isolated murine cardiac fibroblasts were incubated for 15 to 60 min, 24 h or 48 h with 40 µg/ml iE. iE-treatment induces an up-regulation of P-RIP2/RIP2, P-IKK/IKK, P-IκBα/IκBα (15–60 min), NOS2 (24 h) and COX2 (48 h) protein levels. (C) Treatment of fibroblasts with iE for 72 h promoted an increase of TRβ2, P-Smad/Smad, PAI-1 and FGF-2 protein levels. GAPDH was used to normalize all target protein levels. Data are illustrated in histograms as mean±SEM *vs.* vehicle (100%; n = 3–5 samples).*p<0.05, **p<0.01 and ***p<0.001 *vs.* vehicle.

**Figure 7:**
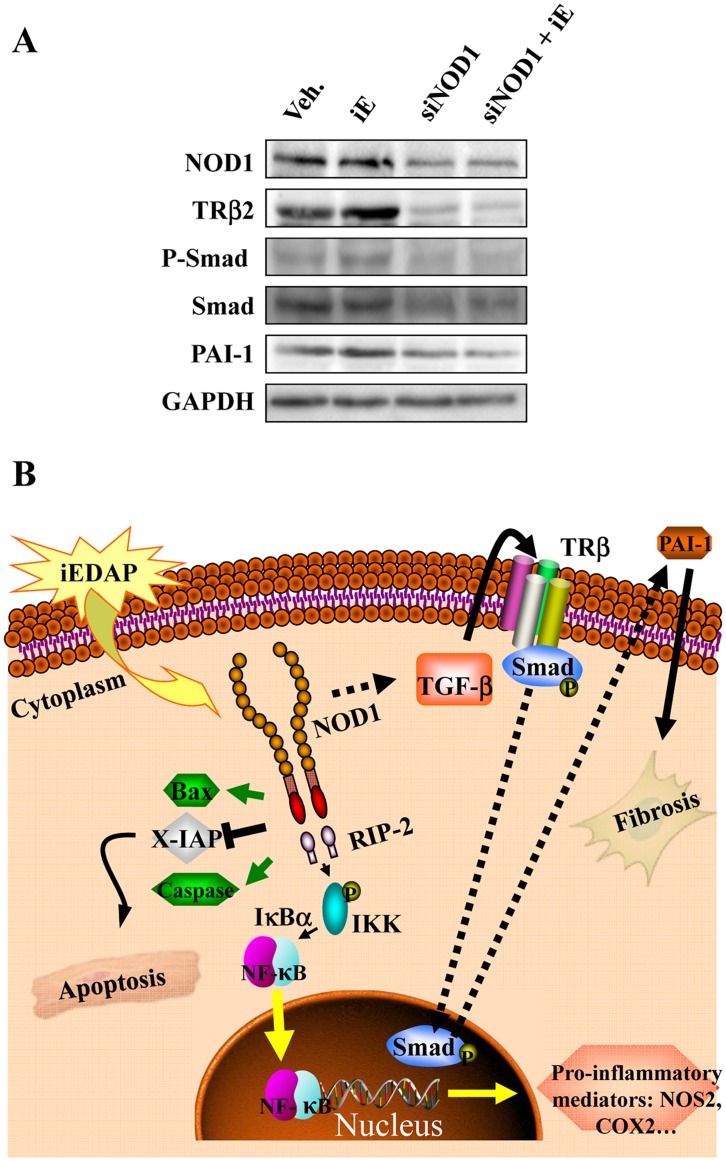
Effect of NOD1 siRNAs on TGF-β pathway. Proposed role for NOD1 activation in cardiac cells. (A) Representative blot of NOD1, TRβ2, P-Smad/Smad, PAI-1, and GAPDH levels in vehicle, iE, NOD1 siRNAs (siNOD1) and NOD1 siRNA + iE (siNOD1+iE) NIH-3T3 cells. Scrambled siRNA sequences did not modify the basal levels after iE-treatment. (B)The selective agonist iEDAP induces NOD1 activation. Both in cardiomyocytes and cardiac fibroblasts, NOD1 promotes the activation of the NF-κB pathway through IKK/IκBα signaling. NF-κB activation induces a proinflammatory response, including the expression of NOS2 and COX2. Moreover, TGF-β is activated in cardiac fibroblasts resulting in PAI-1 transcriptional activation by TRβ and Smad factors. Finally, the enhancement and activation of Bax and caspases and the downregulation of X-IAP promoted by NOD1 agonist in cardiomyocytes, contributes to elicit an apoptotic response associated with NOD1 activation. Altogether, cardiomyocyte apoptosis and the pro-fibrotic profile observed in cardiac fibroblasts might contribute to the onset of cardiomyopathy linked to proinflammatory disease.

### Immunofluorescence Staining

Fibroblasts were seeded onto sterile 8-well chamber slides (Falcon) and fixed with 2% paraformaldehyde for 10 min. Cells were then permeabilized in ice-cold methanol and incubated with 3% BSA for 30 min. After incubating with a rabbit Ab against NOD1 (1∶200), vimentin (1∶200), DDR2 (1∶200) and α-actin (1∶200) (Santa Cruz Biotech. and Invitrogen Corp.) at 4°C over-night, cells were washed with PBS followed by incubation with Alexa 488 anti-rabbit or anti-goat secondary Abs for 1 h at RT (1∶250–1∶500; Molecular Probes). For nuclear counterstaining 4′,6′-diamidino-2′-phenylindoladihydrochloride (DAPI) was used and this was added to the secondary Ab solution in a final concentration of 1 µg/ml. Coverslips were mounted in Prolong Gold antifade reagent (Molecular Probes) and examined using a Leica TCS SP5 spectral confocal microscope. Fluorescence intensity measurements were performed using Image J software (NIH, Bethesda, MD, USA).

### H9c2 and NIH 3T3 Cells

H9c2 (rat cardiac myocyte) and NIH 3T3 (mouse embryonic fibroblast, MEF) cell lines were purchased from the American Type Culture Collection, Rockville. The cell pellets were resuspended in Dulbecco’s medium, supplemented with 10% FBS and antibiotics (penicillin, 100 U/ml; streptomycin, 100 µg/ml). MEFs from IKKβ deficient mice were prepared as described in Methods S1.

### NOD1 siRNA Assays

A pool of 3 target-specific siRNAs or the equivalent scrambled sequences was used to knockdown NOD1 gene expression (Santa Cruz Biotech.). H9c2 and NIH-3T3 cells were transfected with 50 nM of siRNA with Lipofectamine 2000 (Invitrogen) and stimulated with 20 µg/ml of iE. The degree of NOD1 knockdown after 24/48 h of transfection was determined by fluorescein conjugate control of siRNA (Santa Cruz Biotech) and by Western blot analysis. The transfection efficiency of siRNA was 70–80%. Transfection with scrambled RNAs did not modify the NOD1 levels in vehicle and iE-treated cells.

### Cell Death and Viability Detection

For detection and quantification of apoptosis, a TUNEL commercial kit (Roche) was used, following the instructions of the manufacturer. Cell survival assay relies on the capacity of cells to reduce 3-(4,5-dimethylthiazol-2-yl)-2,5-diphenyltetrazolium bromide (MTT) (Calbiochem) to colored formazan in metabolically active cells. Cardiomyocytes suspended in storage solution were incubated for 2 h with 0.5 mg/ml of MTT in the dark at 37°C, and then 100 µl of DMSO was added. Absorbance was measured at 595 nm. All assays were performed per triplicate.

### Histological Analysis

Histological examinations were performed using 3 to 7 animals in each experimental group. Tissue samples were fixed in 10% buffered formalin and embedded in paraffin, and 4-µm sections cut. Hematoxylin/eosin stain was used for analysis to assess morphological changes, and Masson’s trichrome stain was used for detection of collagen. Using a light microscope (Olympus x20) examinations of the slides were performed in a blinded fashion.

### NF-κB Activation Analysis

For detection and quantification of NF-κB activation an ELISA kit (Active Motif, USA) was used, following the instructions of the manufacturer. Absorbance was measured at 450 nm. All assays were performed in triplicate.

### Statistics

Data are presented as means ± SEM. Statistical significance was assessed using a Student´s *t*-test or ANOVA followed by the Bonferroni test when appropriate. Differences with values of p<0.05 were considered statistically significant.

## Results

### NOD1 is Expressed in Heart and its Specific Activation Promotes Cardiac Dysfunction

First, we analyzed NOD1expression by quantitative RT-PCR in different murine tissues. NOD1 was expressed in mouse hearts at similar levels to those other analyzed tissues (Fig. 1A). NOD1 protein levels in mouse hearts were determined by western-blot (Fig. 1B). To determine whether the NOD1-pathway was functional in the heart, mice were treated with the selective NOD1 activator C12-iEDAP (iE) for 2 weeks, and the activation of the NF-κB pathway was investigated. iE treatment promoted an increase in RIP2 phosphorylation, leading to NF-κB activation [26] as deduced by the higher ratios of P-IKK/IKK and P-IκBα/IκBα (Fig. 1B). Also, the binding of nuclear p65 to κB motifs was measured and hearts from iE treated mice showed a significant increase in NF-κB activity (Fig. 1C). Interestingly, hearts isolated from iE-treated mice showed elevated levels of NOS2 and COX2, a response compatible with NF-κB activation (Fig. 1D). From a functional point of view, administration of iE for 2 weeks promoted a time dose-dependent reduction of the ejection fraction, fractional shortening together with a higher systolic volume compared to vehicle treated-mice (Fig. 2A–D, Table 1, and Fig. S1). iE treatment did not affect heart weight (HW) nor body weight (BW) of animals, indicating that this molecule did not induce anatomic cardiac hypertrophy for the period of use (Table 1). The fetal genes associated to cardiac hypertrophy ANP and β-MHC were analyzed by Real–time PCR. Hearts isolated from iE treated mice showed a significant increase in ANP expression compared to vehicle mice-treated ones (20.4 fold induction, p<0.01). β-MHC was not detected in hearts of iE or vehicle treated mice. We speculated that iE mice-treatment might induce a previous step of cardiac hypertrophy, where some fetal genes are activated (ANP) without presenting structural changes. Finally, we treated mice for two weeks with the inactive analogue of iE, iE-Lys and cardiac function was evaluated. Neither cardiac function nor heart weight (HW) or body weight (BW) of mice were modified by iE-Lys treatment (Table 1).

### NOD1 Activation Promotes Cardiac Fibrosis and Apoptosis

It is well established that NOD1 stimulation induces NF-κB activation in different biological systems, and this transcription factor can bind to specific κB sites in the TGF-β promoter, mediating the transcription of this cytokine [27], a major player in the development of fibrosis [28]. Accordingly, we investigated whether NOD1 stimulation induces changes in the pro-fibrotic TGF-β pathway in whole hearts. As Fig. 3A–B shows, an increased deposition of collagen was detected in the hearts of iE treated animals. Moreover, type I collagen mRNA expression was also up-regulated in hearts from iE treated-mice (Fig. 3C). No changes were detected in the expression of type III collagen. In addition to this, iE-Lys (the inactive analogue of iE) did not induce cardiac fibrosis in mice treated for two weeks (Fig. 3A–B). In agreement with these data, the TGF-β/Smad pathway was up-regulated in cardiac tissue from iE-treated mice (2 weeks), showing increased protein levels of TRβ2, P-Smad/Smad and PAI-1–an important target of TGF-β signaling (Fig. 3D). Moreover, the content of the pro-fibrotic mediator fibroblast growth factor 2 (FGF-2) was also elevated in cardiac tissue of treated mice (Fig. 3D). Different reports have demonstrated that NLR proteins cover functions beyond those of the innate immune response, including the regulation of cell death. In this regard, a relationship between NOD1 engagement and caspase activation has been established [15], [29]; therefore, the ability of iE treatment to induce cardiac apoptosis was analyzed using TUNEL assays. Our data show a significant degree of DNA fragmentation in cardiac tissue of iE-treated mice (Fig. 4A) while iE-Lys (negative control for iE) treatment did not induce cardiac apoptotic events (Fig. 4A). Moreover, the levels of pro-apoptotic mediators, such as caspase 3 and Bax were up-regulated, while the anti-apoptotic molecule X-IAP was down-regulated in cardiac tissue from iE-treated mice (Fig. 4B). Together, these data demonstrated that iE administration promotes both cardiac apoptosis and fibrosis and that these changes are accompanied by heart dysfunction.

### NOD1 Stimulation Induces NF-κB and Apoptotic Pathways Activation in Isolated Adult Murine Cardiomyocytes

To further characterize the cardiac changes induced by NOD1 activation, cardiomyocytes isolated from mouse hearts were incubated with iE and different parameters were evaluated. As Fig. 5A shows, NOD 1 was expressed in native cardiomyocytes and NF-κB signaling and their target COX2 expression were significantly higher in iE treated cardiomyocytes. iE-Lys treatment did not induce the expression of the specific adaptor of NOD1, RIP2, or the NF-κB target COX2 (Fig. S2A). To analyze the possible link between NOD1 activation and cardiomyocyte apoptosis, murine cardiac cells were incubated with iE and a dose-dependent loss in cell viability was observed (Fig. 5B). Incubation of cardiomyocytes with staurosporine (Stau) was used as a positive control to induce cell death. On analysis, cardiomyocytes incubated with iE, exhibited higher levels of the pro-apoptotic proteins caspase 3 and Bax, as well as lower levels of the anti-apoptotic proteins X-IAP and Bcl-xL (Fig. 5C). iE-Lys treatment did not modify the caspase 3 and X-IAP protein expression (Fig. S2B). Using the cardiomyocytic cell line H9c2, to avoid the enzymatic isolation process, we demonstrated that NOD1 activation by iE promotes a decrease in cell viability (Fig. 5D) and in X-IAP expression and increases caspase 3 and Bad levels (Fig. S2C) together with NF-κB activation (Fig. S2D). By contrast, NOD1 silencing restored full viability of H9c2 cells treated with iE (Fig. 5D) and reduced caspase 3 activation (Fig. 5E).

### NOD1 Agonist iEDAP Activates NF-κB and TGF-β Pathways in Cardiac Fibroblasts from Adult Mice

Cardiac fibroblasts constitute an important cell population in the heart and are involved in several physiopathological processes, including the development of fibrosis [30]. Cardiac fibroblasts obtained from murine hearts expressed NOD1 (Fig. 6A) and showed higher protein levels of P-RIP2/RIP2, P-IKK/IKK and P-IκBα/IκBα, together with a significant presence of NOS2 and COX2 upon treatment with iE (Fig. 6B). iE-Lys treatment did not promote the expression of the specific adaptor of NOD1, RIP2, or the NF-κB target,COX2, (Fig. S3A). As fibroblasts are the primary source of TGF-β in the heart and might modulate the production of extracellular matrix [31], we analyzed the TGF-β pathway in cardiac fibroblast treated with iE. Our results show that incubation with iE enhances TRβ2, P-Smad and PAI-1 levels as well as higher levels of the pro-fibrotic factor FGF-2 (Fig. 6C). Again, iE-Lys treatment did not modify the expression of the TGF-β target, PAI-1 (Fig. S3B).Taken together, these results suggest a contribution of fibroblasts to cardiac fibrosis and to the NF-κB activity observed in hearts of iE-treated mice. To corroborate the results obtained in native cardiac fibroblasts but avoiding the isolation process, a set of experiments were performed in the murine fibroblast cell line NIH 3T3. In particular, iE treatment of NIH 3T3 induced an up-regulation of NF-κB and TGF-β/Smad/PAI-1 pathways (Fig. S3C–D). Moreover, silencing NOD1 in NIH 3T3 cells blunted the increase in TRβ2 levels, Smad phosphorylation and PAI-1 expression (Fig. 7A) confirming the ability of NOD1 to regulate the TGF-β pathway in response to iE challenge.

## Discussion

It has been well documented that innate immune and inflammatory responses are involved in the pathophysiological processes of myocardial diseases [2]. TLRs and NLRs are pattern recognition receptors that play an important role in the induction of innate immune and inflammatory responses. There is a line of evidence supporting that activation of TLRs contributes to the development and progression of cardiovascular diseases, i.e. atherosclerosis, cardiac dysfunction in sepsis and congestive heart failure [32]. Indeed, the TLR-mediated NF-κB signaling pathway plays a critical role in the induction of innate and immune responses and contributes to myocardial ischemia-reperfusion damage [33], [34], [35], [36]. With respect to NLRs, only limited studies have shown the effect of these pattern-recognition receptors in the cardiovascular field [37], [38], [39]. NOD proteins are NLR-family members implicated in the induction of specific inflammatory responses [11], [12]. Diseases associated with NOD1 activity are mostly chronic inflammatory disorders, e.g., atopic eczema and asthma, underscoring the importance of this receptor in the regulation of the immune response [40], [41], this includes NF-κB activation, cytokine production and, interestingly the induction of apoptosis [14], [42]. In the present report we demonstrated that NOD1 is expressed in whole murine hearts in the range found in other main tissues. We detected a significant protein expression of this NLR in cardiomyocytes and cardiac fibroblasts. A significant finding of the present study is that administration for two weeks of the NOD1 agonist induced profound cardiac dysfunction, together with the activation of the NF-κB and TGF-β pathways and cardiomyocyte apoptosis (see schematic diagram Fig. 7B). Moreover, specific NOD1 stimulation promotes the activation of these pathways in the heart –in both cardiomyocytes and cardiac fibroblasts. iE-Lys, a chemically related molecule to iE but lacking NOD1 activation capacity, failed to exert cardiac dysfunction, pro-fibrotic or apoptotic effects. These results supported the specificity of iE to induce cardiac dysfunction, fibrosis and apoptotic effects in our mice model. Our data provide evidence of functional activation of the classical NF-κB pathway, including phosphorylation of IKK, and the expression of target genes, such as COX2 and NOS2. Moreover, additional experiments performed in mice embryonic fibroblast (MEFs) from wild type and IKKβ-deficient embryos, demonstrated the importance of IKK expression for the ability of iE to induce pro-inflammatory mediators, such as NOS2 (Fig. S4 and Methods S1).

NF-κB activation induced by NOD1 is mediated by the association of the CARD-domain of NOD1 with the corresponding CARD-domain of RIP2 [29], a kinase that mediates immune responses induced by NOD1 and 2 [43]. The relevance of the role of RIP2 in NOD1 signaling is also supported by the analysis of cells derived from mice deficient in RIP2 [29]. Moreover, RIP2 is important for NOD-induced NF-κB activation as well as for apoptotic signaling through its association with members of the TNFR-associated family and members of the inhibitor of the IAP family [44]. Here, we demonstrated that this kinase is activated in whole hearts and in cardiomyocytes and fibroblasts after NOD1 stimulation with iE.

Fibrosis can alter the heart´s structure and architecture with deleterious effects on the cardiac function [45]. In this study, we demonstrated a significant collagen deposition together with a higher expression of type I collagen in hearts of iE treated mice (Fig. 3), this pathological process can induce cardiac dysfunction. TGF-β is a cytokine that has been reported to be a major contributor to tissue fibrosis in various organs [31]. In the heart, TGF-β stimulates the growth of fibroblasts, thus increasing fibrosis. This mediator acts by activating the intracellular pathways linking TGF-β signals to plasma membrane serine/threonine kinase receptors leading in turn to the activation of cytoplasmic effectors such as the Smad proteins. Indeed, phosphorylation of Smad 2/3 and its subsequent translocation to the nucleus are the critical steps in cell signaling through this pathway, leading to collagen deposition in the extracellular matrix. In the heart, profibrotic effects are frequently due to TGF-β-induced fibroblast-dependent increases in extracellular matrix gene expression [31], [46]. In whole hearts, selective NOD-1 activation increases collagen deposition and stimulates the TGF-β/Smad/PAI-1 axis. At the cellular level, iE also induces an up-regulation of this pathway in isolated cardiac fibroblasts and in NIH 3T3 cells. Moreover, we also demonstrated that the knock-down of NOD1 expression by siRNAs efficiently prevented the effects induced by iE on the TGF-β/Smad/PAI-1 pathway. Taken together, our results uncover a novel molecular link between the TGF-β pathway and cardiac NOD1 activation. In agreement with these data, it has been described that NF-κB is bound to specific κB binding sites in the TGF-β promoter, mediating the transcription of this cytokine [27].

Our data identify a relationship between the increase of myocyte apoptosis and NOD1 activation, a response that is exacerbated after iE treatment and prevented by NOD1 interference. NLR proteins are involved in the regulation of cell death, and the presence of a CARD domain in NOD1 confers to these proteins the ability to activate the apoptotic process [29]. In this regard, a connection between NOD1 and caspase activation has been established [15], [29]. Using TUNEL analysis, we observed a greater degree of DNA fragmentation in iE treated cardiac tissue and a decrease in cardiomyocyte viability. Moreover, the levels of pro-apoptotic mediators, such as caspase 3 and Bax were upregulated, whereas the anti-apoptotic protein X-IAP were down-regulated in cardiac tissue and in cardiomyocytes isolated from treated mice. Cell treatment with the specific activator of NOD1 iE was able to induce cell death in a dose-dependent manner and the knock-down of this NLR, by siRNAs, was able to prevent the increase in active caspase 3 protein levels and cell death induced by iE in H9c2 cells. Consistent with our data, Da Silva Correia et al., demonstrated that the NOD1 peptidic ligand, γTriDAP induces apoptosis in MCF-cells [14] and this effect required the expression of NOD1. Some authors described that NOD1 can interact with several pro-caspases containing CARD or DED domains [29], pointing to that this NLR can induce NF-κB activation independently of its ability to promote caspase 9-induced apoptosis.

Our report uncovers a new pro-inflammatory target that is expressed in the heart, NOD1. The specific activation of this NLR can modulate some pathological process such as fibrosis and apoptosis, compromising the cardiac function. All these aspects need to be explored in depth, especially their pathophysiological mechanisms of activation and therefore, their contribution to some cardiac diseases with a pro-inflammatory background, such as heart failure.

Finally, since NLRs seems to be important determinants of human inflammatory disorders and an in-depth understanding of their molecular mechanism of action is required for targeted therapies to be developed. Given this, our study focused on a mice model, shows an increase in inflammatory-mediated up-regulation both in cardiomyocytes and cardiac fibroblasts. Therefore, unraveling the precise contribution of molecules involved in the expression of the inflammatory response not only in professional immune cells, but also in specific organ cells might help to design additional therapeutic interventions and to provide a rationale for identification of new biomarkers for organ dysfunction and targets, such as the NLR system at the cardiac level.

## Supporting Information

Figure S1 **Representative echocardiographic images (M mode) of mouse hearts treated for two weeks with vehicle or C12-iEDAP (iE).**
(DOCX)

Figure S2 **iE-Lys treatment did not modify NF-κB or apoptotic pathways in native cardiomyocytes. iE-DAP, NOD1 agonist induces apoptotic and NF-κB pathways activation in H9c2 cells.**
(DOCX)

Figure S3 **iE-Lys treatment did not modify NF-κB pathway or PAI-1 expression in native cardiomyocytes. Selective activation of NOD1 stimulates NF-κB and TGF-β pathways in NIH-3T3 fibroblasts.**
(DOCX)

Figure S4 **Effect of iEDAP (iE) treatment on MEFs from wild type and IKKβ-deficient embryos.**
(DOCX)

Methods S1 **MEFs culture from wild type and IKKβ-deficient embryos.**
(DOCX)
