# Supplementary material for: NOD1 Activation Induces Cardiac Dysfunction and Modulates Cardiac Fibrosis and Cardiomyocyte Apoptosis
Source: PLoS One. 2012 Sep 18;7(9):e45260. doi: 10.1371/journal.pone.0045260 (PMC3445482; doi:10.1371/journal.pone.0045260)
Supplement: Figure S1 — Representative echocardiographic images (M mode) of mouse hearts treated for two weeks with vehicle or C12-iEDAP (iE). (DOCX) [file pone.0045260.s001.docx]

**SUPPLEMENTAL Figure S1**.

**
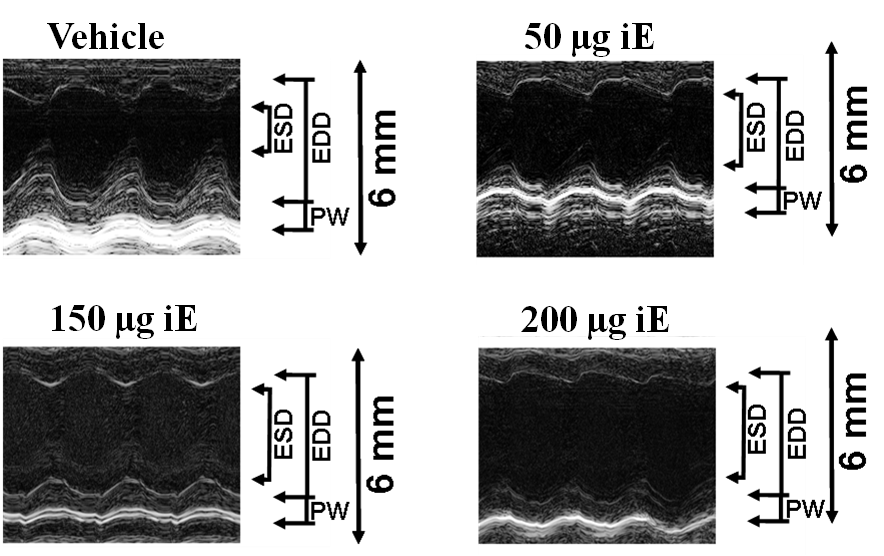
**

**Fig. S1**. **Representative echocardiographic images (M mode) of mouse hearts treated for two weeks with vehicle or C12-iEDAP (iE).** End Systolic Diameter (ESD), End Diastolic Diameter (EDD), and left ventricle Posterior Walls (PW) are indicated.
