# Supplementary material for: NOD1 Activation Induces Cardiac Dysfunction and Modulates Cardiac Fibrosis and Cardiomyocyte Apoptosis
Source: PLoS One. 2012 Sep 18;7(9):e45260. doi: 10.1371/journal.pone.0045260 (PMC3445482; doi:10.1371/journal.pone.0045260)
Supplement: Figure S2 — iE-Lys treatment did not modify NF-κB or apoptotic pathways in native cardiomyocytes. iE-DAP, NOD1 agonist induces apoptotic and NF-κB pathways activation in H9c2 cells. (DOCX) [file pone.0045260.s002.docx]

**SUPPLEMENTAL Figure S2.**


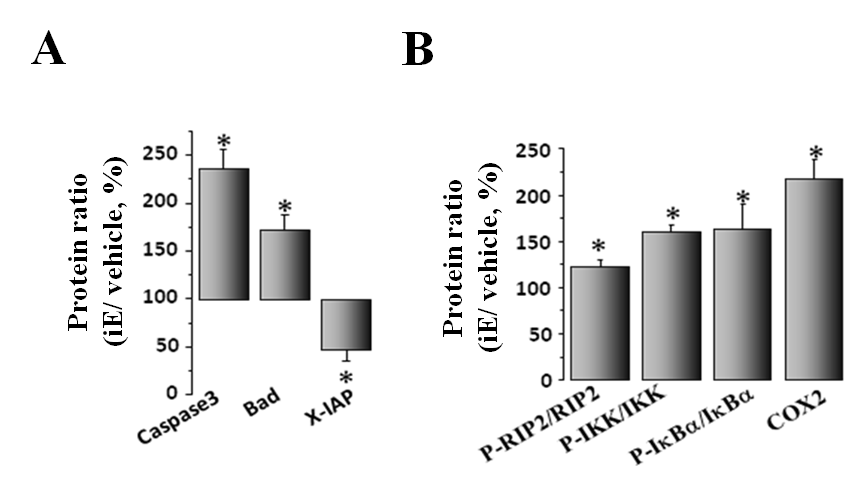

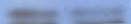


**COX2**

**Veh.**

**iE-Lys**


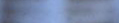


**P-RIP2**


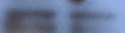


**RIP2**


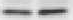


**GAPDH**

**A**


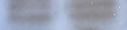


**Caspase 3**

**Veh.**

**iE-Lys**

**GAPDH**

**X-IAP**


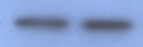

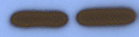


**B**

**C**

**D**

**Fig. S2. iE-Lys treatment did not modify NF-κB or apoptotic pathways in native cardiomyocytes. iE-DAP, NOD1 agonist induces** **apoptotic and NF-κB pathways activation in H9c2 cells.** Representative Western blot of P-RIP2/RIP 2, COX 2 (A), caspase 3 and X-IAP (B) in vehicle and iE-Lys (iE inactive analogue) treated cardiomyocytes . iE-Lys treatment for 15 to 60 min, or 48h with 20 μg/ml did not induce activation of P-RIP2/RIP 2, COX 2, caspase 3 and X-IAP protein levels. H9c2 cells were incubated for 15 to 60 min, or 48h with 20 μg/ml iEDAP (iE). Western blot analysis demonstrated that iE treatment induces an up-regulation of caspase 3, Bad protein expression and down-regulated the X-IAP protein levels (C). iE treatment increased the P-RIP2/RIP2, P-IKK/IKK, P-IκBα/IκBα (15-60 min) and COX2/GAPDH (48h) protein expression (D). Data are expressed as mean±SEM *vs*. vehicle (100%); n=3-5 samples per condition.*p<0.05, *vs.* vehicle.
