# Supplementary material for: NOD1 Activation Induces Cardiac Dysfunction and Modulates Cardiac Fibrosis and Cardiomyocyte Apoptosis
Source: PLoS One. 2012 Sep 18;7(9):e45260. doi: 10.1371/journal.pone.0045260 (PMC3445482; doi:10.1371/journal.pone.0045260)
Supplement: Figure S3 — iE-Lys treatment did not modify NF-κB pathway or PAI-1 expression in native cardiomyocytes. Selective activation of NOD1 stimulates NF-κB and TGF-β pathways in NIH-3T3 fibroblasts. (DOCX) [file pone.0045260.s003.docx]

**SUPPLEMENTAL Figure S3.**


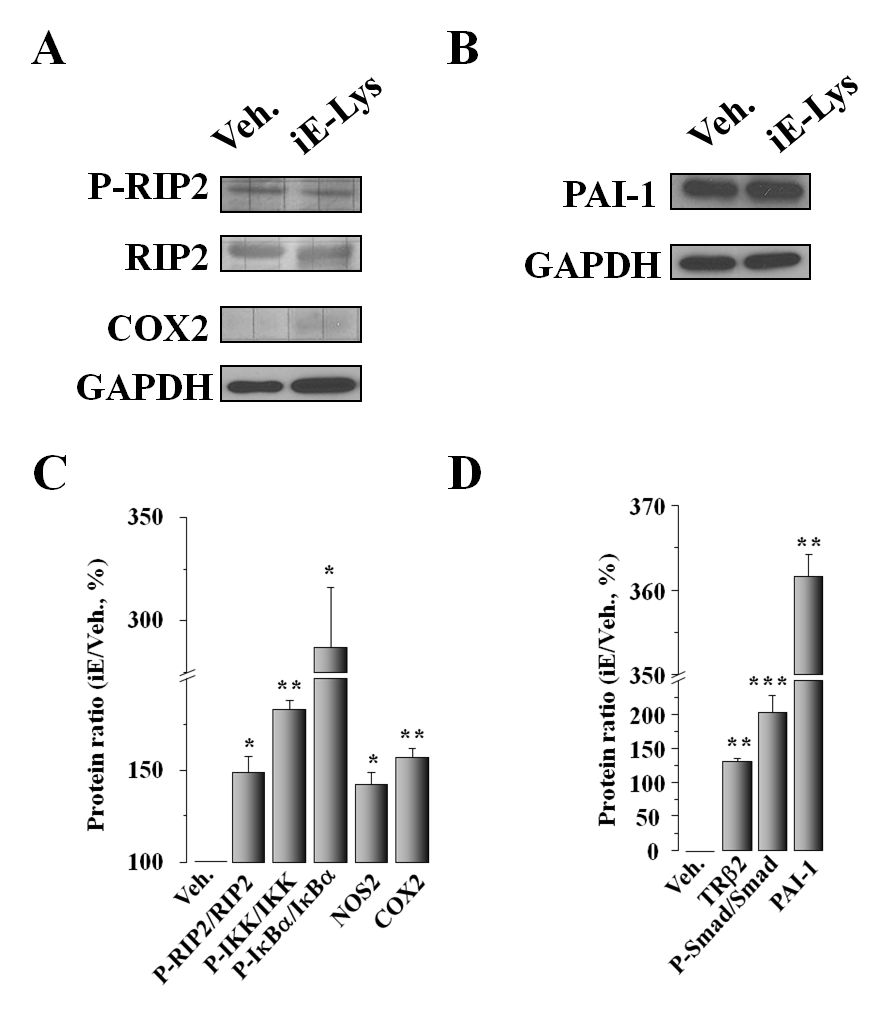


**Fig. S3. iE-Lys treatment did not modify NF-κB pathway or PAI-1 expression in native cardiomyocytes. Selective activation of NOD1 stimulates** **NF-κB and TGF-β pathways in NIH-3T3 fibroblasts.** Representative Western blot of P-RIP2/RIP 2, COX 2 (A), and PAI-1 (B) in vehicle and iE-Lys (iE inactive analogue) treated cardiomyocytes . iE-Lys treatment for 15 to 60 min, or 48h with 40 μg/ml did not induce RIP2 phosphorylation nor changes in the P-RIP2/RIP 2 ratio, nor promoted the expression of COX 2 and PAI-1. NIH-3T3 (C) fibroblasts were incubated for 15 to 60 min, 24 h or 48h with 40μg/ml iEDAP (iE). Western blot analysis demonstrated that iE treatment induces an up-regulation of P-RIP2/RIP2, P-IKK/IKK, P-IκBα/IκBα (15-60 min), NOS2/GAPDH (24h) and COX2/GAPDH (48h) protein levels. Treatment with iE for 72 h promoted in 3T3 cells (D) an increase of TRβ2/GAPDH, P-Smad/Smad and PAI-1/GAPDH protein levels. Data are expressed as mean±SEM *vs.* vehicle (100%; n=3-5 samples).*p<0.05, **p<0.01 and ***p<0.001 *vs.* vehicle.
