# Supplementary material for: NOD1 Activation Induces Cardiac Dysfunction and Modulates Cardiac Fibrosis and Cardiomyocyte Apoptosis
Source: PLoS One. 2012 Sep 18;7(9):e45260. doi: 10.1371/journal.pone.0045260 (PMC3445482; doi:10.1371/journal.pone.0045260)
Supplement: Figure S4 — Effect of iEDAP (iE) treatment on MEFs from wild type and IKKβ-deficient embryos. (DOCX) [file pone.0045260.s004.docx]

**Supplemental Figure S4.**


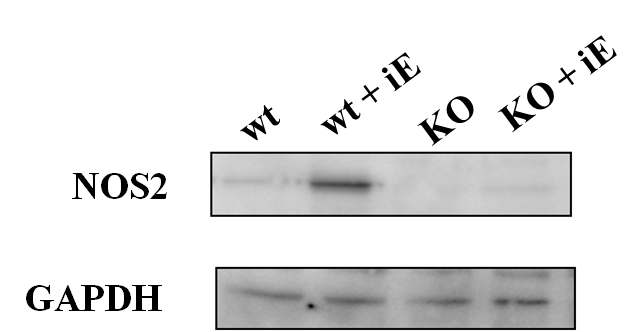


**Fig. S4. Effect of iEDAP (iE) treatment on MEFs from wild type and IKKβ-deficient embryos.** MEFs from wild type and IKKβ-deficient embryos were incubated for 24 h with vehicle or 20 μg/ml iEDAP (iE). Representative blot of NOS2 and GAPDH out of 5 different experiments.
