# Supplementary material for: NOD1 Activation Induces Cardiac Dysfunction and Modulates Cardiac Fibrosis and Cardiomyocyte Apoptosis
Source: PLoS One. 2012 Sep 18;7(9):e45260. doi: 10.1371/journal.pone.0045260 (PMC3445482; doi:10.1371/journal.pone.0045260)
Supplement: Methods S1 — MEFs culture from wild type and IKKβ-deficient embryos. (DOCX) [file pone.0045260.s005.docx]

**SUPPLEMENTAL METHODS**

**Methods S1. MEFs culture from wild type and IKKβ-deficient embryos**. MEFs from wild type and IKKβ-deficient embryos were kindly provided by Dr. A. Bigas (IMIM, Barcelona, Spain). The cell pellets were resuspended in Dulbecco’s medium supplemented with 10% FBS and antibiotics (penicillin, 100 U/ml; streptomycin, 100 μg/ml).
